# Antimicrobial Susceptibility of *Neisseria gonorrhoeae* Isolates in Baltimore, Maryland, 2016: The Importance of Sentinel Surveillance in the Era of Multi-Drug-Resistant Gonorrhea

**DOI:** 10.3390/antibiotics7030077

**Published:** 2018-08-17

**Authors:** Johan H. Melendez, Justin Hardick, Mathilda Barnes, Kathleen R. Page, Charlotte A. Gaydos

**Affiliations:** Division of Infectious Diseases, Johns Hopkins School of Medicine, Baltimore, MD 21205, USA; jhardic1@jhmi.edu (J.H.); mbarnes2@jhmi.edu (M.B.); kpage2@jhmi.edu (K.R.P.); cgaydos@jhmi.edu (C.A.G.)

**Keywords:** *Neisseria gonorrhoeae*, gonorrhea, antimicrobial resistance, azithromycin resistance

## Abstract

The increasing rates of gonorrhea infections and the global emergence and spread of multi-drug-resistant *Neisseria gonorrhoeae* (NG) threaten the successful management of gonorrhea. In the era of nucleic acid amplification tests (NAATs), surveillance projects are urgently needed to monitor prevalence and trends in the antimicrobial susceptibility of NG. In this study, we retrospectively determined the susceptibility profile of NG isolates to previously and currently prescribed antimicrobials. NG isolates collected in Baltimore, Maryland between January and October 2016 were evaluated by the E-test method and/or molecular methods for susceptibility to ceftriaxone, azithromycin, ciprofloxacin, tetracycline, gentamicin, and penicillin. One-hundred and forty-three NG isolates from African-American males (98.6%), primarily heterosexual (88.8%), ranging in age from 15 to 69 years of age were included in the study. Ciprofloxacin resistance was observed in 44.1% of isolates. Plasmid-mediated resistance to penicillin and tetracycline resistance was detected in 22.4% and 10.1% of isolates, respectively. Three isolates (2.1%) displayed high-level resistance to azithromycin (minimum inhibitory concentration (MIC) > 256 μg/mL). Forty-three percent of isolates were resistant or had decreased susceptibility to three antimicrobials (ciprofloxacin, tetracycline, and penicillin). All isolates were susceptible to ceftriaxone and gentamicin. Overall, the epidemiology of antimicrobial resistant NG in Baltimore continues to evolve, and the emergence of azithromycin resistance in this population emphasizes the need for continued sentinel surveillance programs to monitor susceptibility trends and aid in treatment recommendations.

## 1. Introduction

Gonorrhea is the second most commonly reported bacterial sexually transmitted infection (STI) with an estimated yearly incidence of 78 million cases worldwide [1]. *Neisseria gonorrhoeae* (NG) has progressively developed resistance to all commonly prescribed antimicrobials [2]. Due to the high levels of multi-drug-resistant gonorrhea, dual therapy with ceftriaxone and azithromycin is the recommended option [3,4], but NG strains displaying resistance to both of these antimicrobials have now been reported worldwide, threatening the successful treatment of gonorrhea [5].

In order to combat the threat of untreatable gonorrhea, the World Health Organization (WHO) has outlined a series of priorities ranging from the development of new antimicrobials to enhanced surveillance practices to monitor the prevalence and trends of antimicrobial-resistant (AMR) NG [6]. Since the 1980s, antimicrobial resistance trends in the United States have been monitored by the Gonococcal Isolate Surveillance Project (GISP) [7,8]. GISP is a sentinel surveillance program, which collects and analyzes the antimicrobial susceptibility profile of the first 25 NG strains recovered from males from over 25 cities and health departments in the United States. The data generated from this program are then analyzed by the Centers for Disease Control and Prevention (CDC) to make recommendations for the treatment of gonorrhea infections in the United States [3].

In 2016, there were 3534 cases (569.8/100,000) of gonorrhea reported to the Baltimore City Health Department, a 41% increase from 2015 [9]. Because of its high yearly incidence of gonorrhea, Baltimore has traditionally served as one of GISP’s sentinel surveillance sites. However, data regarding the susceptibility profile of NG strains from Baltimore have not been reported since 2013 [10]. In that year, 20.7%, 19.3%, and 4.3% of NG isolates collected for GISP were resistant to penicillin, ciprofloxacin, and tetracycline, respectively, and there were no isolates with decreased susceptibility to ceftriaxone or azithromycin [10]. Considering the high burden of multi-drug-resistant NG and the fast-changing trends of antimicrobial resistance [2], the susceptibility profile of NG isolates from Baltimore should be routinely and continuously monitored to help guide antimicrobial stewardship in this community as well as in the United States.

In the present study, we have retrospectively analyzed NG isolates collected in Baltimore from January to October 2016 to determine the susceptibility profile of these isolates to previously and currently recommended antimicrobials for the treatment of gonorrhea. Additionally, we compare resistance trends in 2016 to the susceptibility data reported by GISP for Baltimore in 2013 to highlight the need for continued and enhanced surveillance practices.

## 2. Methods

### 2.1. Clinical Isolates

Clinical isolates were collected from symptomatic men seeking testing at the Baltimore City Health Department Druid Health Clinic from January to October 2016. Following culture, gram-negative diplococci, oxidase-positive bacterial isolates were presumptively classified as NG, stored in trypticase soy broth with 20% glycerol, and frozen at −80 °C.

In the present study, isolates were recovered by growth on chocolate agar plates incubated overnight in 5% CO_2_ and the colonies re-suspended in phosphate buffered saline (PBS). Two-hundred microliters of each bacterial suspension were extracted for DNA using the automated MagNA Pure LC instrument (Roche Diagnostics, Indianapolis, IN, USA). Following DNA extraction, the isolates were confirmed as NG using a previously described PCR assay targeting the OPA gene [11,12].

### 2.2. Antimicrobial Susceptibility Testing

The E-test method, bioMérieux (Durham, NC, USA), was used to determine the minimum inhibitory concentration (MIC) of ceftriaxone, azithromycin, ciprofloxacin, penicillin, tetracycline, and gentamicin for the NG isolates. Briefly, bacterial suspensions were individually prepared and adjusted to an optical density equal to that of a 0.5 MacFarland standard. Using a sterile swab, bacterial suspensions were then plated on GC agar medium (Becton Dickinson, Sparks, MD, USA) plates supplemented with 1% IsoVitaleX (Becton Dickinson, Franklin Lakes, NJ, USA) and allowed to air dry for 5 min. E-test strips for the six previously described antimicrobials were individually placed on the inoculated agar surface according to manufacturer’s recommendations. All plates were incubated at 36 °C in a moist 5% CO_2_-enriched environment and results recorded after 18–24 h. The MIC was determined by reading the intercept of the inhibition zone and the E-test strip. Breakpoints for antimicrobial resistance (Table 1) were selected in accordance with guidelines set forth by the Clinical and Laboratory Standards Institute (CLSI) [13]. Isolates with MIC ≥ 2.0 µg/mL were classified as azithromycin resistant [14]. Interpretive criteria for gentamicin have not been established by CLSI. Therefore, the breakpoints selected for this study were based on MIC comparison and clinical cure data from previous studies [15,16,17]. The ATCC 49226 and the WHO K and L strains were used as controls during MIC determination. To check for the presence of β-lactamase, all isolates were submitted to the cefinase test [18], and strains with a positive result and penicillin resistance (E-test) were classified as penicillinase-producing NG (PPNG).

### 2.3. Molecular Characterization of Resistance Markers

In order to elucidate the molecular mechanism associated with resistance to fluoroquinolones and penicillin, all isolates were tested with two previously described real-time PCR assays. A PCR assay targeting *GyrA* wild-type sequences was used to detect mutations associated with ciprofloxacin resistance [12,19]. The presence of a plasmid mediating resistance to penicillin was ascertained using a real-time PCR assay [20] as previously described [12].

## 3. Results

### 3.1. Isolate Characteristics and Subject Demographics

**Figure 1:**
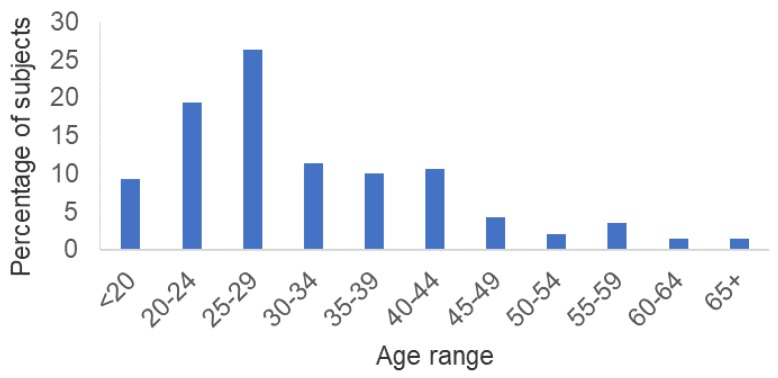
Age range distribution of subjects (*n* = 140).

Of the 172 available isolates, 91.9% (158/172) were viable and of those, 90.5% (143/158) were confirmed by PCR as NG. Fifteen isolates were excluded from further analysis because they were mixed with other bacterial species and isolation of NG was not possible. The 143 NG isolates included in this study were collected from the urethra of 140 subjects ranging in age from 15 to 69 years of age (median = 29) (Figure 1). Three subjects had two isolates each that were collected at two different visits. The majority of subjects (55%) were under 29 years of age. Subjects were predominately (96.8%) African American and heterosexual (88.8%). At the time of gonorrhea diagnosis, 6.6% and 5.8% of subjects were HIV- and syphilis-infected, respectively.

### 3.2. Antimicrobial Susceptibility

**Figure 2:**
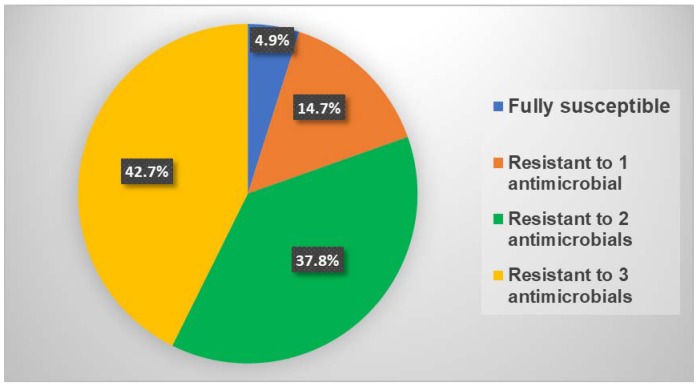
Distribution of NG isolates with resistance or intermediate resistance to multiple antimicrobials.

**Figure 3:**
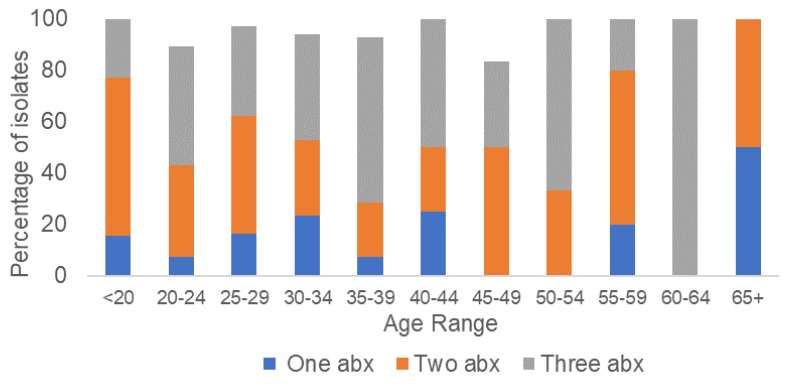
Distribution by age group of NG isolates with resistance or intermediate resistance to multiple antimicrobials. Abx: antimicrobial(s).

All isolates were susceptible to ceftriaxone (MIC range <0.016 to 0.032 µg/mL) and gentamicin (MIC range 1–4 µg/mL). A large proportion of isolates (44.1%) were resistant to ciprofloxacin, while 22.4% and 10.5% of isolates were resistant to penicillin and tetracycline, respectively (Table 1). While the rates of resistance to penicillin (22.4%) and tetracycline (10.5%) were low, this cohort of isolates displayed high rates of intermediate resistance to penicillin (64.3%) and tetracycline (72.7%). Three isolates displayed high-level resistance to azithromycin (AZ-R) (MIC ≥256 µg/mL). The three AZ-R isolates were collected in September 2016 and were susceptible to ceftriaxone, ciprofloxacin, and gentamicin, but displayed intermediate resistance to tetracycline and penicillin. Only 4.9% (7/143) of isolates were susceptible to all of the antimicrobials tested, while 42.7% (61/143) of isolates displayed resistance or intermediate resistance to three antimicrobials (ciprofloxacin, penicillin, and tetracycline) (Figure 2). Seventeen different antimicrobial susceptibility profiles were observed in the 143 isolates tested. The most common resistance phenotype, observed in 25.9% (37/143) of isolates, was ciprofloxacin resistance in combination with intermediate resistance to penicillin and tetracycline. Stratification of the isolates by antimicrobial susceptibility profiles and age group does not suggest an association between age and the acquisition of multi-drug-resistant gonorrhea (Figure 3). However, statistical analysis to evaluate this association was not performed due to the small number of isolates in several of the age groups.

PCR-based molecular characterization revealed that all of the ciprofloxacin-resistant isolates by E-test had (a) mutation(s) in the *GyrA* gene. All of the penicillin-resistant isolates were beta-lactamase-positive and were classified as PPNG based on the presence of the plasmid, which codes for the production of the β-lactamase.

## 4. Discussion

As designated by the WHO in 2012, surveillance of antimicrobial resistance is an important component of the global action plan to control the spread and impact of antimicrobial resistance in NG [6]. In the present study, we report the susceptibility profiles of NG isolates collected in Baltimore, Maryland during a 10-month period in 2016. The data from this analysis will not only help to provide insight into the prevalence of antimicrobial-resistant gonorrhea in Baltimore, but also help to determine how antimicrobial susceptibility trends have evolved over time.

All of the NG isolates analyzed were susceptible to ceftriaxone, which together with azithromycin is recommended as dual therapy for the treatment of gonorrhea. Additionally, a comparison of the ceftriaxone MICs obtained in this study to those reported by GISP in 2013 showed no evidence of increased ceftriaxone MICs in Baltimore. However, high-level resistance to azithromycin was observed in 2.1% of the isolates, which is indicative of the emergence of azithromycin resistance in this population. The observation that all three azithromycin-resistant strains were collected in the same month and that they shared identical MICs to all antimicrobials may be suggestive of the presence of a single clone. Further studies are warranted to determine the clonality of these isolates and to better define the epidemiology of azithromycin resistance in Baltimore and Maryland. The emergence of azithromycin resistance in this population provides further support to the concerns regarding the use of azithromycin in combination with ceftriaxone for the treatment of gonorrhea [21,22].

Although ciprofloxacin is no longer recommended for the treatment of gonorrhea [23], this oral antimicrobial remains a potential option for personalized treatment if susceptibility could be rapidly determined at the point-of-care (POC) [24,25]. In our study, 44% of isolates were resistant to ciprofloxacin, which is higher than the rate (19.3%) reported by GISP in 2013 [10]. However, caution is warranted when comparing the rates observed in 2013 to those obtained in the current study as there were 300 isolates analyzed by GISP in 2013 compared to only 143 in the current study. The levels of resistance to the other previously recommended antimicrobials penicillin and tetracycline were low at 22.4% and 10.5%, respectively. However, the rates of intermediate resistance to these two antimicrobials was high, which has also been recently reported in the first nationwide antimicrobial susceptibility surveillance for NG in Brazil [26].

All of the NG isolates in this study were susceptible to gentamicin. This antimicrobial has been suggested as a useful alternative agent especially in the case of cephalosporin resistance [27]. However, gentamicin has been shown to be more effective for the treatment of uncomplicated gonorrhea when used in combination with azithromycin [28]. In Malawi, gentamicin has been routinely used for the treatment of gonococcal urethritis and the high degree of susceptibility of NG isolates to gentamicin in this country has not changed in more than a decade [15]. Further studies evaluating a larger number of recent NG isolates from different geographical regions are necessary to better define susceptibility-testing guidelines for gentamicin and determine resistance prevalence and trends.

Our study has several limitations. First, the number of samples analyzed in this study was 50% less than the number of samples collected by GISP each year. Second, all the isolates were collected at one clinic, which is predominantly visited by heterosexual African Americans, thus limiting the generalizability and scope of these results. Lastly, we had limited demographical and behavioral data to better define the emergence of azithromycin resistance in this population.

## 5. Conclusions

In conclusion, NG isolates with decreased susceptibility to ceftriaxone were not observed in this study. However, a small number of isolates with high-level resistance to azithromycin were identified, highlighting the need for continued and enhanced antimicrobial surveillance practices. The high degree of susceptibility to gentamicin provides further support for the potential use of this antimicrobial as an alternative option in the event of treatment failure to the first line of defense: ceftriaxone and azithromycin. Lastly, the re-use of the oral antimicrobial ciprofloxacin could be another option to prevent further resistance development especially if susceptibility could be rapidly determined. In the era of nucleic acid amplification tests (NAATs), which prevent the collection of isolates for susceptibility testing, a POC test which could simultaneously identify NG and define antimicrobial susceptibility could not only help to guide antimicrobial stewardship but also be useful for epidemiological purposes.

## Figures and Tables

**Table 1:** Antimicrobial susceptibility profiles of *Neisseria gonorrhoeae* (NG) isolates (*n* = 143).

Antibiotics	MIC Breakpoint (µg/mL) ^a^			Number of Isolates (%)		
	S	I	R	S	I	R
Ciprofloxacin	≤0.06	0.125–0.5	≥1	79 (55.2)	1 (0.7)	63 (44.1)
Penicillin	≤0.06	0.12–1	≥2	19 (13.3)	92 (64.3)	32 (22.4)
Tetracycline	≤0.25	0.5–1	≥2	24 (16.8)	104 (72.7)	15 (10.5)
Azithromycin	≤1		≥2	140 (97.9)		3 (2.1)
Ceftriaxone	≤0.25			143 (100)		
Gentamicin	≤4	8–16	≥32	143 (100)		

MIC: Minimum inhibitory concentration. ^a^ MIC breakpoints were selected in accordance with Clinical and Laboratory Standards Institute (CLSI) guidelines [13] and for gentamicin based on previous studies [15,16,17].
